# Development of a Mobile App to Support Head and Neck Cancer Caregiving: Mixed Methods Study

**DOI:** 10.2196/66471

**Published:** 2025-06-10

**Authors:** Katherine Sterba, Evan Graboyes, Jessica Burris, Megan Scallion, Hannah Kinder, Jama Olsen, Benjamin Toll, Kent Armeson, Terry Day, Bhishamjit Chera, Kenneth Ruggiero

**Affiliations:** 1 Department of Public Health Sciences, Medical University of South Carolina, 86 Jonathan Lucas Street, Charleston, SC, 29425, United States, 1 8438762419; 2 Department of Otolaryngology-Head and Neck Surgery, Medical University of South Carolina, Charleston, SC, United States; 3 Department of Psychology, University of Kentucky, Lexington, KY, United States; 4 Department of Radiation Oncology, Medical University of South Carolina, Charleston, United States; 5 Department of Behavioral Sciences and Psychiatry, College of Nursing, Medical University of South Carolina, Charleston, SC, United States

**Keywords:** head and neck cancer, cancer survivorship, caregiving, nutrition, mobile health, app development, mixed methods

## Abstract

**Background:**

Survivors with head and neck cancer (HNC) face challenging treatment consequences that can lead to severe disruptions in swallowing and result in weight loss, malnutrition, and feeding tube dependence. Caregivers (family or friends who provide support), therefore, often encounter distressing nutritional caregiving burdens and feel unprepared to provide adequate support at home.

**Objective:**

The purpose of this mixed methods study was to develop a mobile support app to support HNC caregiving with an emphasis on nutritional support following treatment.

**Methods:**

We assessed perspectives on nutritional recovery challenges and mobile support app preferences in (1) a national panel of oncology dietitians using a web-based cross-sectional survey and (2) survivors with HNC completing treatment within the past 24 months and their nominated caregivers using dyadic semistructured interviews. Descriptive statistics for survey data were synthesized with thematic analysis of interview data to characterize nutrition-related perceptions and intervention preferences; results were integrated, and themes were translated to high-priority main menu domains and subdomains for a mobile app for caregivers.

**Results:**

Surveys were completed by dietitians (n=116, 100%; female n=87, 50%, with >10 years practice experience). Interviews included survivors with HNC (n=15; 12/15, 80% male, and 6/15, 40% with oropharynx cancer) and their caregivers (n=13; 11/13, 85% female, and 10/13, 77% spouses). Dietitians, survivors, and caregivers perceived that the majority of nutritional concerns assessed (eg, swallowing, feeding tube management, weight maintenance, and caregiver distress about nutrition) were very or extremely important to caregiving in the 6 months following treatment conclusion. The caregiving tasks rated highest in importance by dietitians included tracking nutritional concerns (n=113, 97%), working together as a team on nutritional concerns (n=104, 90%), and making care decisions (n=102, 88%). Five themes emerged from dyadic interviews, including types of nutritional challenges faced, that competing symptoms were difficult to separate from nutritional challenges, the emotional challenges related to nutrition and recovery, the diverse set of medical and support tasks taken on by caregivers, and information and resource needs in caregivers. Qualitative interview and survey themes guided the content of the Healthy Eating and Recovery Together (HEART) app with an intake tracker and sections for nutrition recovery support, other competing caregiving tips, peer support, and caregiver self-care.

**Conclusions:**

Results pinpointed optimal content for a mobile app for caregivers of individuals with HNC and support the acceptability of implementing the HEART app following HNC treatment.

## Introduction

Approximately 522,846 people were living with oral cavity, pharynx, and larynx cancers in the United States in 2021 [1,2]. With increasing numbers of survivors with head and neck cancer (HNC), it is imperative that survivorship concerns are addressed [3,4]. Survivors with HNC face extremely difficult treatment consequences that impair their nutritional well-being [5]. Specifically, HNC and its multimodal treatments [6] can result in oral health problems related to swallowing, speech, mucositis, and dry mouth [7] that disrupt nutritional intake during and after treatment. Most survivors with HNC experience weight loss during treatment [8-11]. Population-level analyses using Surveillance, Epidemiology, and End Results data have estimated that approximately half of patients with HNC need a feeding tube and 40%‐45% of survivors with HNC experience dysphagia-related morbidities up to 2 years following treatment [12]. These nutritional recovery challenges impair the quality of life substantially [13-17].

Nutritional challenges in survivors with HNC also impact their caregivers [18,19], family members, and friends who provide cancer-related support. Relative to caregivers for people with other cancer types, caregivers of people with HNC confront unique support tasks such as feeding tube management, meal preparation, and speech support [6,19-22]. Caregivers report feeling unprepared for their roles in nutritional caregiving, sometimes experiencing a disconnect between survivors’ goals and their own, and experiencing significant unmet needs as caregivers [23-29]. While interventions for caregivers caring for a loved one with cancer have been designed and tested [30-33], few evidence-based interventions are available to support caregivers of people with HNC with a focus on nutritional caregiving tasks in the early posttreatment period. Challenges in providing high-quality comprehensive support to caregivers of people with cancer include cost (financial and time) and competing demands while caring for a loved one [34-36], complexity of survivors’ needs [20], and high prevalence and extent of emotional concerns among both survivors and caregivers [37,38].

Digital health strategies may overcome some of these barriers and offer a promising way to reach and support caregivers of survivors with HNC, particularly during the transition from cancer care to home, a critical point at which in-person interventions may not be feasible [39]. Research is growing on the feasibility, acceptability, and efficacy of digital health interventions for caregivers of people with cancer, with encouraging results for interventions designed to decrease burden and improve mood [39-45]. To guide the development of a mobile app to support nutrition-related caregiving among caregivers of people with HNC, this mixed methods study characterized nutritional challenges and caregiving tasks and intervention preferences in HNC oncology dietitians and survivor-caregiver dyads.

## Methods

### Study Design

Using a concurrent parallel mixed methods design [46], this study included a cross-sectional, web-based survey of a national panel of oncology dietitians and semistructured interviews with survivors with HNC and caregivers. We selected a mixed methods approach to facilitate gathering the perspectives of three groups, including dietitians, survivors with HNC, and caregivers [46]. A mixed methods approach also allowed for a more comprehensive understanding of the unmet needs of caregivers of people with HNC to drive the selection of content in a supportive care app to address those needs. Our team included researchers with expertise and training in HNC, cancer survivorship, oncology caregiving, and mobile health. We used a team approach to reflexivity with discussion and attention to the potential of the research team’s background to influence research findings.

The purpose of this mixed methods study was to characterize nutritional challenges and caregiving tasks and intervention preferences in HNC oncology dietitians (quantitative) and survivor-caregiver dyads (qualitative and quantitative). We concurrently collected survey data from dietitians. Both quantitative and qualitative data were considered of equal priority and were analyzed separately and then integrated using the merging technique [47] as described below. Data were collected between April and September 2018. The GRAMMS (Good Reporting of A Mixed Methods Study) checklist was used to guide the mixed methods approach and reporting [48] (Checklist 1). Multimedia Appendix 1 provides the interview guide and surveys.

### Dietitian Surveys

A convenience sample of dietitians was recruited by posting a study notice on the listserv for the Oncology Nutrition Dietetic Practice Group of the Academy of Nutrition and Dietetics. Dietitians were eligible for the 15-minute web-based survey if they reported providing care for patients with HNC in the past 6 months; the survey was hosted on REDCap (Research Electronic Data Capture; Vanderbilt University). The 67-item survey was developed by the study team. All items were optional, and participants could return to the survey over time if requested. The survey assessed demographic (race, ethnicity, sex, and age) and practice (credentials, years practicing as a dietitian, practice setting, and number of patients with HNC seen per week) characteristics. In addition, perceptions of the importance of posttreatment concerns in caregiving (0=not at all important to 4=extremely important) and perceived importance and difficulty (0=not important or difficult at all to 4=extremely important or difficult) of a variety of support tasks for caregivers of people with HNC, guided by the transactional model of caregiving were assessed (eg, tracking nutritional intake, changes and patterns in symptoms, and making care decisions [49]). Other measures included ratings of nutritional support resource needs (0=not at all to 4=extremely) in caregivers of people with HNC (eg, screening process to identify caregiver nutritional concerns, assessment tool to identify caregiver distress, and educational materials) and barriers to addressing support needs (1=not a barrier at all to 4=major barrier) in caregivers of people with HNC (eg, time, caregiver interest, lack of evidence about the value of caregiver programs, and leadership). Participants then reviewed example app screens and then answered questions about the preferred focus of app content (eg, increasing caregivers’ awareness of the importance of addressing nutritional challenges, changing caregivers’ attitudes about improving nutritional status, and encouraging help-seeking for nutritional support) using an adapted version of the app-specific subscale in the Mobile App Rating Scale [50] (1=strongly disagree to 6=strongly agree) and an open-ended question. Finally, participants completed an open-ended question asking them to describe any specific suggestions they had for the development of a mobile support app for HNC caregivers.

### Dyad Interviews

Survivors with HNC who completed treatment with curative intent 6‐24 months prior to enrollment and were free of disease, and their caregivers, were recruited at the Medical University of South Carolina Hollings Cancer Center, with initial screening for eligibility by chart review. Inclusion criteria included being 18 years or older, completing treatment for stage I-IVA HNC (mucosal squamous cell carcinoma of the oral cavity, oropharynx, hypopharynx, and larynx), and experiencing nutritional challenges at the end of treatment as assessed by a 6-item screen. Survivors were excluded if they were unable to identify a primary caregiver, and survivors and caregivers were excluded if they either did not speak English or were cognitively impaired. After mailing a study letter and determining eligibility via phone screen, dyadic interviews were scheduled. Informed consent documents were signed, and dyadic interviews were conducted in person by 2 female investigators with training in qualitative methods (KS and MS). Interviewers did not know the participants and were not involved in their clinical care. Interviews were continued until we reached saturation in themes [51]. They were conducted in a private room, audio-recorded, and lasted approximately 45 minutes. Field notes were taken to provide interview observations.

We developed a semistructured interview guide using a cancer survivorship quality of life framework [52] to examine participants’ reflections on the physical, emotional, and social challenges they experienced at the end of treatment and in the 6 months following treatment conclusion, with an emphasis on nutritional challenges and caregiving. Survivors and caregivers were asked to describe their emotional and physical well-being at the end of treatment. They were asked specifically about nutritional challenges, expectations they had about intake abilities, and the caregiver’s role in nutritional recovery. Finally, they were asked about caregiver needs and suggestions for resources to better meet their needs at the end of treatment. Participants then viewed a set of example app screens (eg, nutrition tips, recipes, and stress management advice) on a tablet, after which they provided feedback on the content and format of a future app. After the interview, survivors and caregivers completed separate brief paper-based surveys assessing demographic (age, race, sex, and education), clinical (stage, treatment type, and nutritional status at the end of treatment), and technology access (home computer and smartphone) characteristics. They also completed ratings of the importance of caregiving tasks (0=not important to 4=extremely important) and ratings of agreement (1=strongly disagree to 6=strongly agree) about the benefits of checking in with caregivers after treatment, providing support messages to caregivers, and the importance of providing practical information to help with patients’ nutritional recovery.

### Ethical Considerations

Study procedures were approved by the Medical University of South Carolina Institutional Review Board (Pro00066211). A waiver of written informed consent was granted for dietitian surveys; after reading a study statement, participants advancing to the survey implied consent. Dyads completed written informed consent before completing interviews and received a copy of the signed consent for their records. All screening and survey data were stored in password-protected databases. The underlying databases were hosted in a secure data center. All data were identified only by code number (participant IDs). Dietitians completing surveys were entered into a lottery to receive a US $25 gift card to thank them for their time. Survivors with HNC and caregiver participants each received a US $50 gift card.

### Statistical Analysis and App Development

Descriptive statistics were used to characterize survey data on dietitian and survivor-caregiver dyad demographic characteristics, perspectives about posttreatment, and caregiving challenges, and app preferences using R (R Core Team) [53]. Interviews were transcribed and analyzed using rigorous content analysis methods for systematic theme identification [54] in NVivo software (QSR International) [55]. Transcripts were coded by pairs of independent coders (KS, JO, and HK) and regrouped and reorganized until the investigators agreed on categories. This initial inductive theme identification process was followed by team meetings to finalize themes and guide implications for the intervention design. We sought trustworthiness in the qualitative data analysis approach by including prolonged engagement with the data, triangulating data, and using an audit trail to finalize themes [56]. Quantitative and qualitative data were analyzed separately, and then a data synthesis integration step was used to guide app development [57]. We selected the mixed methods integration approach of merging [47] and brought our quantitative and qualitative results together for elaboration. For example, the quantitative results (eg, perceptions and preferences of dietitians, survivors, and caregivers) were merged with key themes identified in interviews used to offer a more in-depth understanding of appropriate content for the app. Our interdisciplinary team of HNC clinicians, researchers, and developers completed a set of planning meetings to translate study results into app content by discussing the meaning of themes, identifying potential similarities and differences across themes, and mapping the themes to content using a consensus-based approach. The app development method was an agile approach, specifically rapid application development or rapid application building, which focuses on timely delivery in a fast-paced environment with the use of prototyping and iterative development [58]. The research team worked closely with the development team to review and test evolving prototypes; a final prototype was pretested with 2 caregiver volunteers who were not involved in interviews or development activities.

## Results

### Participant Characteristics

#### Dietitians

All dietitians (n=116, 100%) were female, and the majority were registered dietitians (n=115, 99%) and White (n=110, 95%). Most practiced more frequently in the outpatient (n=107, 92%) versus inpatient (n=23, 20%) setting; options not mutually exclusive. Half had more than 10 years of experience working with patients with HNC, and most (n=87, 75%) cared for 1‐10 patients with HNC per week.

#### HNC Dyads

A total of 50 survivors mailed a study letter. Several (n=15, 30%) were ineligible due to exclusion criteria, while others declined (n=20, 40%) due to scheduling conflicts prohibiting attendance at the in-person interview, lacking interest or reporting being too ill, or overwhelmed to participate. In total, 15 survivors enrolled in the study and nominated a caregiver. All survivors completed the in-person interview and 11 survivors were accompanied by their caregivers (8 spouses and 3 children). Scheduling conflicts precluded interview completion for 4 caregivers in person, 2 of whom were interviewed independently by phone (n=13 caregivers overall).

Most survivors (n=12, 80%) were male, while most caregivers (n=10, 77% spouse) were female (n=11, 85%). A total of 83% (n=19) of survivors and 87% (n=20) of caregivers were White, and age varied from 28 to 79 years (mean age 66, SD 15.1 for survivors and 61, SD 16.6 for caregivers). A total of 20% (n=3) of survivors and 27% (n=3) of caregivers had a high school or lower education. The most common cancer types included oropharynx (n=6, 40%) and oral cavity (n=3, 20%). Most survivors had surgery (n=14, 93%) and radiation (n=12, 80%); not mutually exclusive. One-third (n=5, 33%) had a liquid diet and approximately half (n=7, 47%) had a feeding tube at the end of treatment. Finally, most participants had a home computer (n=13, 87% survivors; n=13, 100% caregivers) but fewer survivors than caregivers had a smartphone (n=11, 73% vs n=10, 90%).

### Posttreatment Challenges

#### Perceptions of HNC Nutritional Concerns in Caregiving

**Figure 1.**
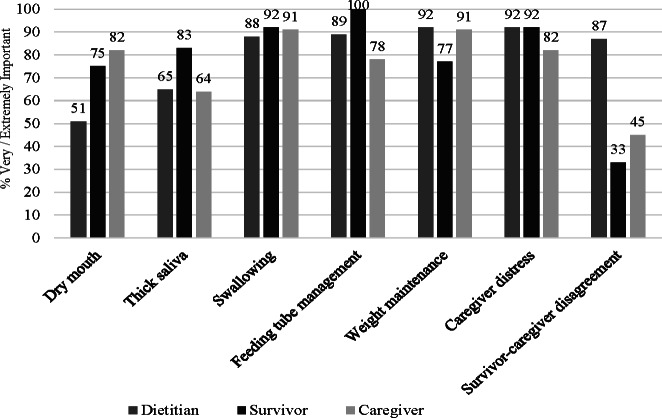
Dietitian, survivor, and caregiver perceptions of the nutritional concerns in caregiving after treatment. This figure reports data from dietitian (n=116), survivor (n=15), and caregiver (n=11) surveys. Each caregiving concern endorsed at the very or extremely important level is graphed for dietitians, survivors, and caregivers.

In the 6 months following treatment completion, dietitians, survivors, and caregivers perceived that the majority of nutritional concerns assessed were important to caregiving (Figure 1). The caregiving concerns rated most important (ie, very or extremely) by dietitians included weight maintenance (n=107, 92%), feeding tube management (n=103, 89%), caregiver distress about nutrition (n=103, 89%), and swallowing (n=102, 88%). The caregiving concerns rated as most important by survivors included feeding tube management (n=8, 100%), swallowing (n=14, 92%), and caregiver distress about nutrition (n=14, 92%). The caregiving concerns rated most important by caregivers themselves included weight maintenance (n=10, 91%), swallowing (n=10, 91%), caregiver distress about nutrition (n=9, 82%), and dry mouth (n=9, 82%).

#### Perceptions of HNC Caregiving Tasks

Most dietitians perceived all evaluated caregiving tasks to be very or extremely important to survivors’ recovery. The highest-rated caregiving tasks included tracking nutritional concerns (n=113, 97%), working together as a team on nutritional concerns (n=104, 90%), and making care decisions (n=102, 88%). Relative to importance, most dietitians did not perceive that the measured caregiving tasks were overly difficult for caregivers to perform. The caregiving tasks most frequently endorsed as very or extremely difficult included navigating the health system (n=45, 39%), making care decisions (n=44, 38%), and interpreting changes in nutritional status (n=40, 34%).

#### HNC Recovery Challenges

Dyadic interviews identified 5 unique themes, which are described below with example quotes from survivors and caregivers shown in Textbox 1.

Textbox 1. **Themes identified in interviews with survivors with head and neck cancer and their caregivers.**

**Nutritional challenges were expected but extremely frustrating with weight loss and disrupted intake caused by texture/taste, swallowing, dry mouth and sticky saliva concerns**

Exemplary survivor quotes:

- Well, the issue for me is that, I mean I couldn't taste food and water tasted really weird. So I basically ate because I knew I was supposed to. Not because I enjoyed it.
- [My wife] said, “Baby, please eat, eat something.” Cause I mean, I'm doing my best...I'm trying, I'm trying, I'm trying, and I'm trying but I wish I could just eat something.
- Peg tube blockages [happened because] protein powder didn’t dissolve well, gurgling, irritation. Part of lining of my wound started sticking out and ached. After hospital discharge, I was given a nutrient solution from a medical supply company and then had diarrhea, night sweats, and projectile vomiting. Lost 26 pounds.

Exemplary caregiver quotes:

- I kept weighing him and you know in the back of your mind you know that losing weight is a sign of cancer and you really, in your mind you know it's gone, you think it's gone. But there's that worry that maybe it's not really gone and that's why his weight is still going down. So that really confused me about his weight.
- But what bothered me was that he won't eat or couldn't eat and he lost a lot of weight. He looked terrible, and he didn't have energy. I didn't know what to fix when we get home. I tried to do mostly liquid stuff but there's only so much liquid stuff you can do. The thing is, they had pulled all of his molars before the surgery, because they figure if he had a radiation, it would have to have his teeth fit. Well, with no teeth, you can't chew.
- It did affect him quickly and he dropped the weight like a ton of bricks. It was a challenge to get anything down him and it was like I wanted to do things on his schedule but it was just really, really difficult. We tried this and we tried that and we tried you know, tried several different things.

**Other competing symptoms were common and difficult to separate from nutritional challenges**

Exemplary survivor quotes:

- Well, the pain had a lot to do with that. Your neck, shoulders, arms and hands are in lots of pain and discomfort.
- When I finished everything, I think the last two weeks for me were the worst, where the radiation had really taken on where it had in my jaw because I could probably open my mouth the width of my finger.
- I had two really bad sores in my mouth that didn't make it fun to eat anything. When I started again occasionally, I would get some irritation from eating crunchy foods.
- I couldn't raise my arms up above my shoulders… I think my taste was coming back and the physical therapy really, I mean, that really turned me around. I started feeling a lot better. They really rehabbed my shoulders.

Exemplary caregiver quotes:

- And the sores that was on his neck kind of bothered me. Some nights he couldn't sleep normally. You know, you just go to sleep but he couldn't.
- It was more helplessness than anything. There's not anything you can do for him. You can't force him to eat because the pain was there.
- His stomach was upset a lot. After the radiation was over for a period of time after the radiation was over. It was just miserable. And also he is having [GI] issues.
- But as far as some of the other issues as far as being nauseous, we had to deal with that. The doctor gave her medicines for that, another big one is constipation. Oh, that one is a tough one.

**Emotional challenges related to nutrition and recovery were common**

Exemplary survivor quotes:

- Oh, yeah, confused, I mean the cancer just takes the world, just takes a lot out of yourself and I wondered if I'm going back to work and how I'm going to feel at the end of the day, for how long I would be able to see my brand new granddaughter.
- I'm still at the point where anything - I'm still afraid…because you know I still do have a lump down there, I know every time I swallow my saliva I know it’s there and I can feel it and it's - that's one thing that's always going back of my mind.
- Emotionally, I was depressed especially after the radiation specifically. Physically, I couldn't swallow. I couldn't eat. It was the most egregious treatment I ever had my life and if I knew what the outcome was going to be I would have never done it.

Exemplary caregiver quotes:

- I was with her through her depression. I think, I was depressed along with her. Seeing my mother not being able to eat or any of that and on Sundays we have Sunday dinners at her house so, we went ooh, about a good two, three months without having Sunday dinner at mom's unless one of us had to go over and cook.
- Depression lingered, it's always in the back of your mind - is it coming back? Or did they get it all? Or those kinds of things and then what's going to be next?
- At the end of treatment I was a basket case. I checked on him constantly when he slept.

**Caregivers take on a diverse set of medical and support tasks**

Exemplary survivor quotes:

- Well my wife made up a chart and we had everything, I want to say laminated, definitely a plastic cover over it which was a good thing. We had it all laid out there [to monitor intake].
- But I mean-- patience. Because we're going to fight back, not meaning to be mean. But we're hurting. We're just trying to get back—we’re trying to get better. I would just say, frustration. That'd be a better word. Because it is frustrating. And you seem to take it out on the ones you love the most.
- I know I relied on them a lot to get me and to help me get to the tub, to get me out of the tub. At that time, it wasn't easy. If I was this big, they wouldn't have been-- it would take a couple of them.

Exemplary caregiver quotes:

- I went to the grocery store and I just went up and down the aisle trying to find something that he could eat.
- You wouldn’t believe what I’ve learned to do from the last year. Changing IV’s. Trained to do those things. There’s nothing I wouldn’t do for him anyway, but I was nervous I would maybe do something wrong and harm him accidentally.
- Knowing that he's like a glass half empty kind of guy, I tried to keep everything - I didn't... not that I didn't think about it, but I just kept thinking positive. That they got it all.
- You need someone who have their best interest at heart. There's times that she's not in her right state of mind with everything that is going on so, we go to these doctor’s appointments or we go to certain things, you have to have someone that is there that's going to ask the doctors the question.

**Caregivers need information about what to expect with nutritional challenges and recovery**

Exemplary survivor quotes:

- [My caregiver] was given a calorie intake document for a day and that was what we're trying to keep at, but there's no real specifics given us - as to how we should accomplish that.
- Yeah, I think seeing a nutritionist would have been a plus. That was never approached to us. Maybe while I was in the hospital. Given some kind of program to try to keep certain calories or what kind of foods and how to build up to.
- I think that we should have been counseled about … a feeding tube. There was no plan. There was no nutritional plan. So, full disclosure and a plan to support that disclosure would have been great. And early on in the planning – you need it in the treatment planning phase.

Exemplary caregiver quotes:

- It would be nice to have a list of do's and don'ts, a list of things that have worked for patients in the past like, you know. Puddings, Jell-O's, more common-sense and anything but having a list would be really nice when you're in a grocery store and going, okay, he's gonna need blank, blank, blank and blank.
- Well, I think they should give you a folder with all the instructions for about everything. Nutrition, food to eat, everything that needs to be done on our way. Or even we didn't know you had to have this port flushed.
- Okay, I think what could be helpful...it seems to me, if somebody can be pre-briefed as to what difficulties they may have with the-- like the chewing aspects and the saliva aspects, and what things that people have found in general that might be worth avoiding, that would be a helpful thing.

##### Theme 1: Nutritional Challenges Were Expected but Extremely Frustrating With Weight Loss and Disrupted Intake Caused by Texture or Taste, Swallowing, Dry Mouth, and Sticky Saliva Concerns

Both survivors and caregivers reported an array of nutritional challenges at the end of treatment that required special diets and feeding tubes. With challenges in swallowing, texture, and taste during recovery, dyads reported difficulty finding diets that were satisfying and tolerable, and often caregivers experienced distress in encouraging their loved ones to eat. Shopping, cooking, and communicating and negotiating with one’s loved one around intake were also commonly reported challenges. Weight loss was reported by all survivors and caused significant distress, particularly in caregivers. The routine around feeding was reported to be very tedious.

##### Theme 2: Other Competing Symptoms Were Difficult to Separate From Nutritional Challenges

Survivors experienced many symptoms in addition to and related to nutritional concerns. For example, participants reported fatigue, mobility challenges, nausea, sores, and pain, and recovery challenges that often exacerbated nutritional and caregiving concerns. The array of symptoms caused frustration in survivors and worry in caregivers.

##### Theme 3: Emotional Challenges Related to Nutrition and Recovery Were Common

The emotional challenges faced by survivors and caregivers included frustration, fear, uncertainty, confusion, and depression. They reported frustration associated with eating challenges and the persistent focus on feeding and symptom management. Some participants reported worry and fear about survivors not regaining functional abilities. Relatedly, confusion and uncertainty about the future were commonly described by both survivors and caregivers. Caregivers expressed fear about weight loss and choking, as well as their ability to care for their loved ones. Both survivors and caregivers reported depression and other psychosocial concerns, and both also emphasized the importance of caregiver well-being.

##### Theme 4: Caregivers Take on a Diverse Set of Medical and Support Tasks After Treatment Completion

Caregivers focused on practical, nutritional, and emotional support tasks. Common practical tasks included providing transportation to and attending health care appointments, helping a loved one get around, and managing medications and stoma and IV care. Common nutritional support tasks included monitoring weight loss and intake, grocery shopping, researching recipes, preparing meals, and caring for feeding tubes. Emotional support tasks included supporting survivors’ frustration with recovery and nutritional challenges and trying to keep a positive attitude. It was common for caregivers to report distress about support challenges, and they sometimes faced resistance from their loved ones around eating. Caregivers described a dynamic process of being persistent, creative, and patient.

##### Theme 5: Caregivers Need Information and Resources About What to Expect and How to Cope With Nutritional Challenges

Caregivers described feeling unprepared to support nutritional recovery and said they would have benefited from additional resources and support at the end of treatment. They emphasized the importance of early education during the treatment planning process to provide a better understanding of what to expect, resources and tools to support food preparation, and tips to help monitor intake. Survivors and caregivers both highlighted interest in meeting with a dietitian, yet also raised concerns about information overload.

### Caregiver Needs and App Recommendations to Support Caregivers

Dietitians endorsed caregivers’ time constraints (100/116, 86%) and caregivers being overwhelmed (102/116, 88%) as major barriers to meeting caregivers’ needs. They also endorsed oncology clinics lacking designated staff to coordinate caregiver resources (70/116, 60%), higher priority care issues (n=72/116, 62%), and clinical team time constraints (n=70/116, 60%) as barriers. In light of unmet needs and to better support caregivers’ provision of quality nutritional support to survivors with HNC, dietitians rated the importance of a variety of services and resources. While all 8 proposed services were rated by the majority (≥60%) of dietitians as very or extremely important, the highest ranked services or resources included a clinic referral process to link caregivers to appropriate nutritional resources (104/116, 90%), educational materials about diet (92/116, 79%), one-on-one counseling about nutrition (103/116, 89%), and training in nutritional support and symptom management (90/116, 78%).

Dietitians’ responses to an open-ended question after reviewing example app screens yielded suggestions for new app content, including recipe recommendations, intake tracker, encouragement about caregiving tasks, and support for the caregiver’s own well-being. Exemplary quotes include:

*Caregivers want recipes! Tips for sore mouths, mucositis and dry mouth* ... *trouble-shooting enteral tube issues, constipation tips.*

*Specific tips on adding high-calorie foods for weight maintenance (low in acid, soft/liquid). Same for high protein foods for tissue healing and muscle mass maintenance/recovery. Tips for frequent eating until appetite improves or side effects diminish (nausea, early satiety).*

*Having exercises listed that can maintain muscle strength would be great. Also, a tracking device for the number of tube feedings completed and fluid intake would be helpful.*

*It is important for the caregiver to have one set number to call for info. So many times, they try to get info from the internet which isn’t always helpful.*

*Support for caregivers themselves knowing they are not alone and such a big part of success moving forward.*

Survivors and caregivers’ survey responses indicated that dyads were in strong agreement that checking in with caregivers after survivors complete treatment (12/15, 80% and 9/11, 82%, respectively) and providing support messages to caregivers (11/15, 73%, and 10/11, 91%, respectively) would be helpful. In addition, dyads were in strong agreement that it is important to provide practical information to caregivers to help survivors’ nutritional recovery (15/15, 100%, and 10/11, 91%, respectively).

Feedback from dyads’ responses to open-ended questions after reviewing example app screens included keeping the content simple, providing tips about what foods to avoid, providing recipes and dynamic nutrition information as needs change, including tips from other survivors with HNC and caregivers, and emphasizing support for caregiver well-being and self-care. Examples of exemplary quotes include:

*This is like your personal resource right here at your fingertips - you’re not alone. Let them know you’re here if they need you. Simplicity is important*.

[Caregiver]

*Provide more advice about foods you can eat and problems with specific types of food groups…practical information with step-by-step directions. Food suggestions based on symptoms*.

[Survivor]

*Prepare caregivers for the possibility that the patient may not like the same dishes caregivers prepared before and not to take this personally*.

[Caregiver]

*Encourage caregivers to ask for support from others: Doesn’t mean you’re less of a person [if you ask for help]. You need all the help you can get and a lot of the time you don’t want to ask for it*.

[Caregiver]

*It is important to ask about how the caregiver is doing; I feel guilty for not asking how she was doing. In general, we are in the dark; anything that brings some light into the room is helpful*.

[Survivor]

### Data Synthesis: App Development

Building on results from all surveys and interviews underscoring the high need and interest in a comprehensive caregiver app, we designed the Healthy Eating and Recovery Together (HEART) app. The overall goal of the app was to support caregivers of survivors with HNC as they transition to the home setting after completing treatment and decrease their unmet needs and caregiver burden. The study investigators evaluated qualitative themes from dyad interviews side by side with quantitative themes observed in dietitian and dyad surveys. Team meetings were used to identify similarities in themes that were translated to high-priority main menu domains and subdomains for the app. The integration of survey and qualitative data resulted in an expansion of the findings, as the qualitative themes provided a detailed understanding of quantitative findings about caregivers’ unmet needs and responsibilities as caregivers. The research team worked closely with the development team to review and test evolving prototypes, and a final prototype was pretested with 2 caregivers. The app was updated with feedback over the course of these steps (eg, we updated icons, modified menu choices, added instructions to components, reordered messages, and modified color choices). HEART includes educational information, caregiving tips and encouragement, and resources, with 4 elements, including survivor nutritional support, intake tracker, caregiver toolkit, and support videos (Table 1 provides more detailed descriptions and sample screenshots). The app also provides caregivers with notifications twice a week to check in with them about their concerns and deliver real-time resources mapped to current concerns. Two main areas in the app’s content included a focus on survivor-caregiver teamwork and support of caregivers’ own well-being.

**Table 1.** Content of the HEART^a^ app.

App section	Content	Results guiding content	Screenshot
Nutritional support	Tips and encouragement for supporting a loved one with nutritional intake	A need for coverage of a broad array of topics to support the dynamic nutritional recovery process with content on common issues, recipes, and oral care support.	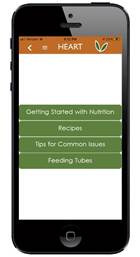
Intake trackers	Supports tracking of feeding tube, liquid diet, and solid food in real time to monitor quantity, tolerability, and preferences, with the ability to share the intake journal with others.	Caregivers experience distress in monitoring intake and need a simple, convenient way to support monitoring.	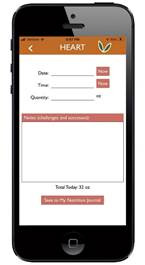
Caregiver toolkit	Emphasis on caregiving tasks and how caregivers can take care of their own physical and emotional well-being with tips and relaxation techniques	Caregivers feel unprepared for their roles as caregivers and face significant burdens, often overlooking their own well-being.	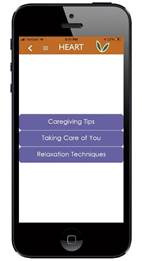
Support videos	Survivor, caregiver, and clinician videos to support nutritional recovery and well-being	Interest in social support and increased interaction with dietitians and other survivors and caregivers.	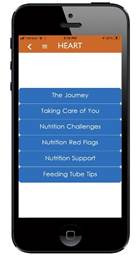
My resources	Stores biweekly prompts and tailored resources	Participants desire real-time connections and resources that are dynamically matched to their changing needs during recovery and caregiving.	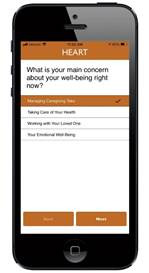

a HEART: Healthy Eating and Recovery Together.

## Discussion

### Principal Results

Survivors with HNC and their caregivers face exceptionally difficult posttreatment concerns that negatively impact their quality of life [28,59-61]. Caregivers are often tasked with addressing survivors’ nutritional challenges in the home setting, yet many are unprepared for these caregiving tasks [20,24]. Getting adequate nutritional intake, maintaining a healthy weight, and managing physical symptoms and emotional distress are imperative for survivors of HNC during the posttreatment period; yet, there is a paucity of tools available to support survivors and their caregivers in meeting these goals [31,32]. This study addressed this important survivorship care gap, specifically the availability and accessibility of high-quality interventions to support nutritional caregiving. Recognizing the value of stakeholder input in intervention development [62-64], we used mixed methods to assess the perspectives of survivors, and then also used responses from caregivers and oncology dietitians to guide the development of an app to support caregivers at the end of treatment. Previous studies have emphasized the acceptability of apps for cancer caregivers while also calling for more formative research to ensure their suitability [43,44,65,66]. While we intended to focus this tool on nutritional caregiving support, study results led to a more comprehensive product focused on nutrition and other survivor recovery concerns, plus caregiver self-care. The final HEART app includes nutrition support with an intake tracker, along with tips and encouragement for other caregiving areas (daily support, emotional support, and medical support), support videos from peers and clinicians, and a caregiver toolkit (taking care of yourself and relaxation exercises).

To guide app development, dietitians provided their perspectives on caring for and supporting HNC dyads. They endorsed a broad array of important nutritional concerns for HNC caregiving, including swallowing, feeding tube management, weight maintenance, and caregiver distress about nutrition. They also perceived that there were multiple nutritional care tasks that were important to survivors’ recovery, including monitoring nutrition, making care decisions, and working together with their survivors as a team to manage nutritional concerns. While most dietitians did not rate these care tasks as extremely difficult for caregivers to manage, the number of tasks endorsed was substantial, and the importance to survivors’ recovery was rated highly, indicating that an app intervention would likely need to be complex and cover a comprehensive set of nutritional concerns. These results suggest that an app should help caregivers be flexible and skilled in multiple nutritional care tasks. Additionally, dietitians’ perceptions regarding potential survivor-caregiver mismatch on nutritional goals were consistent with previous studies [18,26], which may suggest that a focus on teamwork in dyads would be beneficial in an app [67,68].

HNC survivor-caregiver dyads in this study confirmed the challenges reported by dietitians in both surveys and interviews. First, survey results highlighted similar nutritional caregiving concerns after treatment in survivors, caregivers, and dietitians. Second, interviews confirmed the types of nutritional challenges experienced and the emotional toll they take on dyads, consistent with previous studies [5,6,69,70]. Results also highlighted that nutritional caregiving tasks were compounded by other competing HNC recovery concerns (eg, support for pain, mobility, pain, fatigue, and emotional challenges); it was difficult for caregivers to focus on nutrition without considering these additional concerns. As highlighted in previous research, caregivers take on a multitude of caregiving tasks and need resources matched to these responsibilities [14,19,20], again supporting the coverage of a broad array of HNC caregiving [20] content in an app.

Dietitians also endorsed high barriers to meeting caregiver needs, including clinician time constraints and a lack of designated staff to coordinate resources. While increasing research has prioritized interventions to meet caregiver needs [31,71], cancer care settings are not adequately resourced to address their needs [72,73]. Technology tools such as the HEART app may be a promising approach to address these barriers and complement other interventions to support caregivers [39,74]. Dietitians in this study endorsed a broad variety of strategies and content to include in an app to support caregivers. Dietitians tended to prefer high-resource strategies for inclusion in an app, such as intake tracking, training, screening processes, educational resources, and counseling. Survivors and caregivers confirmed interest in an app for caregivers with check-ins, support, and practical tips. Both dietitians and dyads recommended the provision of recipes and support for caregivers’ own well-being. Dietitians uniquely recommended an intake tracker, while dyads uniquely recommended tips from peers.

While mobile health intervention development and testing to support caregivers of people with cancer is growing and shows evidence of promising acceptability, adherence, and some improvements in short-term outcomes such as caregiver burden [41-43,45,65,75,76], more research is needed in this area to better understand caregiver adoption and engagement in these interventions, impacts on caregiver psychosocial and health outcomes, and best practices for disseminating such intervention in practice using rigorous methods. Of key importance, few studies have focused on HNC caregiving and nutritional support. Apps to support caregivers of people with HNC also have the potential for supporting and addressing HNC dietitian time constraints by supporting their recommendations outside of the clinic; more research is needed in this area.

### Strengths and Limitations

The strengths of this study include its mixed methods approach with data collection from multiple perspectives, including those who had experienced HNC recovery, served as an HNC caregiver after treatment, and provided clinical care for HNC survivor-caregiver dyads. The integration of survey data and qualitative data allowed for the examination of similarities and unique findings across qualitative and quantitative themes, ultimately allowing a deeper understanding of perspectives to guide the HEART app. Dietitians were recruited from across the United States, and qualitative interviews with dyads were used to supplement surveys and provide a more in-depth understanding of survey results. The app’s focus on nutritional support, particularly after the end of treatment, is innovative and would help address an area of major concern for HNC providers, survivors, and caregivers. In the context of changing digital health use patterns over time, it is important to note that an important limitation of this study is that the data were collected in 2018. However, it is notable that digital health use and engagement rates have increased over time, and this expanded reach and growing societal acceptance of these tools are therefore encouraging [77]. Other limitations of this study include recruitment of dyads from only one medical center, recruitment of a convenience sample of dieticians, and a modest sample size for interviews, all of which limit the transferability of findings. In addition, we experienced a lack of diversity in clinical and sociodemographic factors for surveys and interviews. We selected a parallel convergent approach for data collection and analysis and followed with an integration approach (merging) to synthesize our mixed methods data; while a sequential approach may have allowed more iterative app development and testing, this approach was selected to facilitate rapid technology development.

### Conclusions

In summary, this study identified the optimal content for a mobile support app for caregivers of people with HNC and supported the acceptability of implementing this intervention at the end of treatment. Future steps include evaluating the implementation of the HEART app and its impact on survivor and caregiver outcomes. It will be important for a future study to rigorously test the HEART app in a prospective clinical trial.

## Supplementary material

10.2196/66471 Multimedia Appendix 1 Study Interview Guide and Suveys.

10.2196/66471 Checklist 1 GRAMMS (Good Reporting of A Mixed Methods Study) checklist.

## Abbreviations

GRAMMS: Good Reporting of A Mixed Methods Study
HEART: Healthy Eating and Recovery Together
HNC: head and neck cancer
REDCap: Research Electronic Data Capture
