# Supplementary material for: Development of a Mobile App to Support Head and Neck Cancer Caregiving: Mixed Methods Study
Source: JMIR Cancer. 2025 Jun 10;11:e66471. doi: 10.2196/66471 (PMC12172808; doi:10.2196/66471)
Supplement: Multimedia Appendix 1 [file cancer-v11-e66471-s001.pdf]

Oncology Dietitian Questionnaire

You are being invited to participate in the Healthy Eating and Recovery Together (HEART) study at the Medical University of South Carolina.

Your participation in this study will involve completing a one-time survey. This survey should take approximately 10 minutes to complete. Your participation will be confidential and you will not be contacted to complete any other study activities. This survey involves minimal risk to you. You will have the option of being entered in a gift card drawing for a $25 Amazon gift card to thank you for your participation.

The goal of this survey is to assess oncology dietitians' perspectives on the nutritional support challenges faced by close family members (caregivers) who are supporting a loved one with head and neck cancer. Your responses will help us learn more about the nutritional challenges faced by those supporting a loved one with head and neck cancer and create a mobile support tool to address these challenges.

Your participation in this survey is completely voluntary. You may choose not to complete the survey. You do not have to answer any question for any reason. If you have questions about this study or if you have a research-related problem, you may contact the study coordinator at 843-876-2438 or Dr. Katherine Sterba, the investigator in charge of the study, at 843-876-2419.

For questions about rights as a research participant, you can contact the Medical University of South Carolina Institutional Review Board for Human Research IRB Manager or the Office of Research Integrity Director at 843-792-4148.

The completion of this survey implies your consent to participate. If you choose to participate, please first complete the following screening question below to confirm you are eligible. Thank you!

Do you currently provide (or have you previously in the last 6 months provided) care for head and neck cancer patients as a dietitian?

☐ No: Thank you for your willingness to participate. Unfortunately, this study is for dietitians who care for patients with head and neck cancer so you are not eligible to participate.
☐ Yes: Great, please begin the survey.

A. Nutritional Support Tasks for Family Members Supporting a Patient with Head and Neck Cancer

How IMPORTANT do you believe the following support tasks are for a family or friend caring for a loved one with head and neck cancer ***in the first 6 months after completing treatment***? Please select one answer for each item.

Items	Response Options				
	**Not at all Important**	**Slightly Important**	**Moderately Important**	**Very Important**	**Extremely Important**
1. Tracking nutritional intake	0	1	2	3	4
2. Tracking patient nutritional concerns (e.g., swallowing problems, distress about eating)	0	1	2	3	4
3. Interpreting the frequency, changes and patterns in nutritional symptoms	0	1	2	3	4
4. Making care decisions and adjustments as needed (e.g., adjusting food timing/amount, modifying diet consistencies, planning ahead)	0	1	2	3	4
5. Accessing nutritional support resources	0	1	2	3	4
6. Providing hands-on care (e.g., food preparation, feeding tube care)	0	1	2	3	4
7. Working together with patient to address nutritional concerns	0	1	2	3	4
8. Navigating health system for better nutritional care	0	1	2	3	4
9. Other: please specify: ____________________________	0	1	2	3	4

Thinking more about these caregiving tasks, how DIFFICULT do you believe each task is for the family caregiver to carry out ***in the first 6 months after completing treatment***?

Items	Response Options				
	**Not at all**	**Slightly**	**Moderately**	**Very**	**Extremely**
1. Tracking nutritional intake	0	1	2	3	4
2. Tracking patient nutritional concerns (e.g., swallowing problems, distress about eating)	0	1	2	3	4
3. Interpreting the frequency, changes and patterns in nutritional symptoms	0	1	2	3	4
4. Making care decisions and adjustments as needed (e.g., adjusting food timing/amount, considering support consequences, planning ahead)	0	1	2	3	4
5. Accessing nutritional support resources	0	1	2	3	4
6. Providing hands-on care (e.g., food preparation, feeding tube care)	0	1	2	3	4
7. Working together with patient to address nutritional concerns	0	1	2	3	4
8. Navigating health system for better nutritional care	0	1	2	3	4

B. Caregiver Concerns

Please rank the following head and neck cancer nutritional concerns for caregivers on a scale from 1 (most important) to 8 (least important).

_____ Dry mouth

_____ Thick saliva

_____ Feeding tube management

_____ Swallowing

_____ Weight maintenance

_____ Transition from enteral nutrition to oral intake

_____ Caregiver distress related to patient's nutritional status

_____ Disagreement between patients and their caregivers about nutritional status

C. Caregiver Nutritional Support Resources

To what extent do you believe the following resources are needed to support family caregivers to provide quality nutritional support for patients with head and neck cancer ***in the first 6 months after completing treatment***?

Items	Response Options				
	**Not at all**	**Slightly**	**Moderately**	**Very**	**Extremely**
1. A screening process integrated in the clinic to identify caregiver nutritional concerns.	0	1	2	3	4
2. An assessment tool in place to identify caregiver distress about the patient's nutritional status.	0	1	2	3	4
3. A clinic referral process to link caregivers to appropriate nutritional resources.	0	1	2	3	4
4. A dedicated information line for caregivers to call when needing nutritional information.	0	1	2	3	4
5. Printed educational materials about diet for distribution to caregivers.	0	1	2	3	4
6. A list of online nutritional support resources for caregivers.	0	1	2	3	4
7. One-on-one counseling for caregivers about nutrition.	0	1	2	3	4
8. A symptom reporting tool for caregivers to report patient nutritional symptoms remotely.	0	1	2	3	4
9. Training for caregivers in nutritional support and symptom management.	0	1	2	3	4
10. Support groups for caregivers focused on nutritional support and symptom management.	0	1	2	3	4
11. Video-conferencing with oncology dietitians and caregivers to provide nutritional support.	0	1	2	3	4
12. Other? Please specify: ____________________	0	1	2	3	4

D. Nutritional Support Tool for Caregivers

We are developing a mobile support tool to monitor concerns and provide information, tips and videos to caregivers of head and neck cancer patients at the end of treatment. This mobile support system (or "App") will prompt caregivers twice a week to report any concerns they have about their loved one's nutritional status and link them to information and encouraging messages. Please review the examples of screenshots below and then complete the rating questions that follow. **[show screenshots]**

Thinking about a mobile support system (or "App") for head and neck cancer caregivers, please select one number that best describes how strongly you agree or disagree with the statements below about the system.

	Strongly Disagree	Moderately Disagree	Slightly Disagree	Slightly Agree	Moderately Agree	Strongly Agree
**An App for head and neck cancer caregivers should focus on…**						
1. Increasing caregivers' awareness of the importance of addressing nutritional challenges.	1	2	3	4	5	6
2. Changing caregivers' attitudes toward improving nutritional status.	1	2	3	4	5	6
3. Increasing caregivers' intentions / motivation to address nutritional challenges.	1	2	3	4	5	6
4. Encouraging help-seeking for nutritional support.	1	2	3	4	5	6
5. Providing a process to link caregivers to needed resources.	1	2	3	4	5	6
6. Improving caregivers' distress about patient nutritional challenges.	1	2	3	4	5	6

Do you have any suggestions (topics to cover, areas of support to provide) as we develop a mobile support tool for head and neck cancer caregivers?

____

E. Barriers to Caregiver Service Delivery

Listed below are several barriers some clinicians have noted may adversely impact assessing and addressing the nutritional <u>support needs</u> of head and neck cancer <u>family caregivers</u>. Please indicate the extent to which you believe each factor impacts service delivery for <u>caregivers</u> at your practice.

Items	Response Options			
	**Not a barrier at all**	**Minor barrier**	**Moderate barrier**	**Major barrier**
1. Caregivers do not have enough time	☐	☐	☐	☐
2. Lack of caregiver interest	☐	☐	☐	☐
3. Caregivers are too overwhelmed	☐	☐	☐	☐
4. Not enough time for clinical team	☐	☐	☐	☐
5. Other issues are higher priority to clinical team	☐	☐	☐	☐
6. Lack of available educational resources for caregivers	☐	☐	☐	☐
7. Privacy concerns regarding sharing patient information with caregivers	☐	☐	☐	☐
8. Availability of dietitian	☐	☐	☐	☐
9. Lack of evidence about the value of caregiver programs	☐	☐	☐	☐
10. Lack of administrative leadership	☐	☐	☐	☐
11. Lack of designated staff to coordinate resources	☐	☐	☐	☐
12. Inadequate IT systems for documentation	☐	☐	☐	☐
13. Inadequate communication among clinical team members	☐	☐	☐	☐
14. Lack of reimbursement for staff to coordinate education and counseling services	☐	☐	☐	☐
15. Other: please specify ________________________________	☐	☐	☐	☐

F. Characteristics

We have a few final questions about you and your practice.

1. What are your credentials? Please check all that apply.

☐ RD
☐ CSO
☐ CNSC
☐ Other: please specify ____

2. During a typical week, approximately how many **head and neck cancer patients** do you see in your primary practice location?

☐ 0
☐ 1-10
☐ 11-20
☐ 21-30
☐ 31-40
☐ 41-50
☐ > 50

3. How many years have you been practicing as a dietitian?

☐ less than 1 year
☐ 1-5 years
☐ 6-10 years
☐ 11-20 years
☐ > 20 years

4. In what type of setting do you practice? Please check all that apply.

☐ Outpatient
☐ Inpatient
☐ Other: please specify ____

5. Are you of Hispanic origin or ancestry?

☐ No
☐ Yes

6. Which do you feel best describes your race or ethnicity? Please check all that apply.

☐ American Indian/Alaska Native
☐ Asian
☐ Native Hawaiian or other Pacific Islander
☐ Black or African-American
☐ White

7. What is your current age?

- ☐ 20-30 years old
- ☐ 31-40 years old
- ☐ 41-50 years old
- ☐ 51-60 years old
- ☐ > 60 years old

8. What is your gender?

- ☐ Male
- ☐ Female

Thank you for completing this survey. If you would like to be entered into a drawing for an amazon gift card, please provide your name and email address here: ______________. Please note that your survey responses will not be linked to your name. We appreciate your commitment to helping develop programs to support the nutritional recovery of head and neck cancer patients.

Please complete a few questions before your interview. All answers are confidential and will only be used to describe the people who participate in our interviews. Thank you!

Information about Your Health

When were you first diagnosed with head and neck cancer?

Month: ________ Year: ________

What was the stage of your cancer diagnosis?

❒ Stage I
❒ Stage II
❒ Stage III
❒ Stage IV
❒ Other: please specify ________________________________
❒ Not sure or don't know

What treatment did you receive for your cancer? (Please check all that apply.)

❒ Surgery
❒ Chemotherapy
❒ Radiation
❒ Immunotherapy
❒ Other: please specify ________________________________
❒ Not sure or don't know

Since you were first diagnosed with head and neck cancer, did a doctor or other healthcare professional tell you that your cancer had come back?

❒ No ❒ Yes

If yes, please describe:

____
____
____
____
____
____

<u>Your Thoughts About the HEART Program</u>

We are planning a program focused on helping family caregivers to support their loved ones with head and neck cancer at the end of treatment. For this program, patients and caregivers will complete a set of questions using tablet computers and caregivers will receive a care plan along with mobile-support for one month.

The first questions are about your opinions about using the tablet computer to answer questions about your needs and concerns.

**How comfortable would you be…**	Not at all Comfortable	A Little Comfortable	Somewhat Comfortable	Very Comfortable	Extremely Comfortable
Using the tablet computer to answer questions?					
Reading the questions on the tablet computer?					
Following the instructions for each question?					
Holding the tablet?					
Moving from one question to the next?					

When you think back about your recovery after completing head and neck cancer treatment, please rate how important you believe the following nutritional concerns were for your caregiver.

	Not at all Important 1	2	3	4	Extremely Important 5
Your dry mouth					
Your thick saliva					
Your feeding tube management					
Your swallowing					
Your weight maintenance					
Your caregiver's distress about your nutritional status					
Disagreement between you and your caregiver about your nutritional status					

Are there any other caregiver concerns or needs you believe it is critical we address at the end of treatment?

____

____

Please think about your nutritional intake at the end of all of your treatment. Please read through each of the choices and check ALL items that applied to you at the end of treatment.

At the end of treatment, I was taking:

- ❒ *Normal food (the same type of food you were taking prior to your cancer diagnosis)*
- ❒ *Normal food* but less than normal amount
- ❒ Little solid food
- ❒ Mostly or only liquids
- ❒ Nutritional supplements
- ❒ Very little of anything

At the end of treatment, how satisfied were you with your nutritional status?

❒ Not at All Satisfied ❒ A Little Bit Satisfied ❒ Somewhat Satisfied ❒ Very Satsified ❒ Extremely Satisfied

What have been your biggest __nutritional__ recovery challenges with cancer since you completed treatment?

____
____
____
____

Now please think about the care plan example we showed you. Please read each item below and circle one number to tell us how much you agree or disagree with the statement.

The information provided in the care plan would be helpful to patients and families emotionally.

1	2	3	4	5	6
Strongly Disagree	Moderately Disagree	Slightly Disagree	Slightly Agree	Moderately Agree	Strongly Agree

The information provided in the care plan would be practical to help caregivers provide support to their patients.

1	2	3	4	5	6
Strongly Disagree	Moderately Disagree	Slightly Disagree	Slightly Agree	Moderately Agree	Strongly Agree

Now please think about the mobile support part of the program (messages, videos and tips) for caregivers. Please read each item below and circle one number to tell us how much you agree or disagree with the statement.

Checking in with caregivers after their loved ones complete treatment would be helpful.

1	2	3	4	5	6
Strongly Disagree	Moderately Disagree	Slightly Disagree	Slightly Agree	Moderately Agree	Strongly Agree

Providing support messages to caregivers after treatment would be helpful.

1	2	3	4	5	6
Strongly Disagree	Moderately Disagree	Slightly Disagree	Slightly Agree	Moderately Agree	Strongly Agree

It is important to provide practical information to caregivers to help with patients' nutritional recovery.

1<br>Strongly<br>Disagree	2<br>Moderately<br>Disagree	3<br>Slightly<br>Disagree	4<br>Slightly<br>Agree	5<br>Moderately<br>Agree	6<br>Strongly<br>Agree

Do you have any suggestions to improve our HEART mobile support tool?

____
____
____

We plan to hold the HEART clinic session after patients finish all of their treatment. Thinking back to when you completed treatment, please think about when you and your caregiver would have benefited from the HEART session in your cancer care.

❒ Before completing my treatment
❒ Right at the end of all of my treatment
❒ Around 1 month after finishing all of my treatment
❒ Around 3 months after completing all of my treatment
❒ Around 6 months after completing all of my treatment

Information about You

This information will be used to describe the people who participated in our interviews.

What is your age? _________

What is your gender? ❒ Male ❒ Female

Are you of Spanish/Hispanic origin? ❒ Yes ❒ No

What is your race? (Please check all that apply.)

❒ White
❒ Black or African American
❒ American Indian or Alaska Native
❒ Native Hawaiian or Other Pacific Islander
❒ Asian (please specify group): __________
❒ Other (specify): ______________________

What is your current employment status? (Please check all that apply.)

❒ Full-time paid work
❒ Part-time paid work
❒ Homemaker
❒ Unemployed
❒ Receiving Disability
❒ Retired
❒ Other: please specify: ____

What is your current health insurance status?

❒ Private Insurance
❒ Medicaid
❒ Medicare
❒ Self-pay out of pocket
❒ No insurance
❒ Other: ____________________

What is your marital status?

❒ Single
❒ Married
❒ Widowed
❒ Separated/Divorced
❒ Have a current partner

What is the highest level of schooling that you completed?

❒ Less than 12 Years
❒ High School Graduate or GED
❒ Technical or trade school
❒ Some College
❒ College Graduate
❒ Graduate School

Do you have a computer at home with daily access to the internet?

❒ No ❒ Yes

Do you have a smart phone or tablet that connects to the Internet, such as an iPhone, Blackberry, or Droid?

❒ No ❒ Yes

Thank you!

HEART STUDY
Study ID:
Group ID:
Date:

Your Thoughts About the HEART Program

We are planning a program focused on helping family caregivers to support their loved ones with head and neck cancer at the end of treatment. For this program, patients and caregivers will complete a set of questions using tablet computers and caregivers will receive a care plan along with mobile-support for one next month.

The first questions are about your opinions about using the tablet computer to answer questions about your needs and concerns.

**How comfortable would you be …**	Not at all Comfortable	A Little Comfortable	Somewhat Comfortable	Very Comfortable	Extremely Comfortable
Using the tablet computer to answer questions?					
Reading the questions on the tablet computer?					
Following the instructions for each question?					
Holding the tablet?					
Moving from one question to the next?					

HEART STUDY
Study ID:
Group ID:

When you think back about your loved one's recovery after completing head and neck cancer treatment, please rate how important you believe the following nutritional concerns were for you as a caregiver.

	Not at all Important 1	2	3	4	Extremely Important 5
Your loved one's dry mouth					
Your loved one's thick saliva					
Feeding tube management for your loved one					
Your loved one's swallowing					
Your loved one's weight maintenance					
Your distress about your loved one's nutritional status					
Disagreement between you and your loved one about his/her nutritional status					

Are there any other caregiver concerns or needs you believe it is critical we ask at the end of treatment?

____

____

At the end of your loved one's treatment, how satisfied were you with his/her nutritional status?

❒ Not at All Satisfied ❒ A Little Bit Satisfied ❒ Somewhat Satisfied ❒ Very Satsified ❒ Extremely Satisfied

What have been your biggest challenges with your loved one's nutritional recovery since he or she completed treatment?

____

____

____

____

Now please think about the care plan example we showed you. Please read each item below and circle one number to tell us how much you agree or disagree with the statement.

The information provided in the care plan would be helpful to support families emotionally.

1<br>Strongly<br>Disagree	2<br>Moderately<br>Disagree	3<br>Slightly<br>Disagree	4<br>Slightly<br>Agree	5<br>Moderately<br>Agree	6<br>Strongly<br>Agree

The information provided in the care plan would be practical to help caregivers provide support to their patients.

1<br>Strongly<br>Disagree	2<br>Moderately<br>Disagree	3<br>Slightly<br>Disagree	4<br>Slightly<br>Agree	5<br>Moderately<br>Agree	6<br>Strongly<br>Agree

Now please think about the mobile support part of the program (messages, videos and tips) for caregivers. Please read each item below and circle one number to tell us how much you agree or disagree with the statement.

Checking in with caregivers after their loved ones complete treatment would be helpful.

1<br>Strongly<br>Disagree	2<br>Moderately<br>Disagree	3<br>Slightly<br>Disagree	4<br>Slightly<br>Agree	5<br>Moderately<br>Agree	6<br>Strongly<br>Agree

Providing support messages to caregivers after treatment would be helpful.

1<br>Strongly<br>Disagree	2<br>Moderately<br>Disagree	3<br>Slightly<br>Disagree	4<br>Slightly<br>Agree	5<br>Moderately<br>Agree	6<br>Strongly<br>Agree

It is important to provide practical information to caregivers to help with patients' nutritional recovery.

1 Strongly Disagree	2 Moderately Disagree	3 Slightly Disagree	4 Slightly Agree	5 Moderately Agree	6 Strongly Agree

Do you have any suggestions to improve our HEART mobile support tool?

____
____
____

We plan to hold the HEART clinic session after patients finish all of their treatment. Thinking back to when your loved one completed treatment, please think about when you would have benefited from the HEART session in your loved one's cancer care.

- ❒ Before completing his/her treatment
- ❒ Right at the end of all of his/her treatment
- ❒ Around 1 month after your loved one finished all of his/her treatment
- ❒ Around 3 months after your loved one completed all of his/her treatment
- ❒ Around 6 months after your loved one completed all of his/her treatment

Information about You

This information will be used to describe the people who participated in our interviews.

What is your age? _________

How are you related to your loved one who has cancer?

❒ Spouse ❒ Child (Daughter or Son) ❒ Brother or sister
❒ Partner ❒ Other Relative: ______________________________
❒ Parent ❒ Other: ____________________________________
❒ Friend

What is your gender? ❒ Male ❒ Female

Are you of Spanish/Hispanic origin? ❒ Yes ❒ No

What is your race? (Please check all that apply.)

❐ White
❐ Black or African American
❐ American Indian or Alaska Native
❐ Native Hawaiian or Other Pacific Islander
❐ Asian (please specify group): __________
❐ Other (specify): ______________________

What is your current employment status? (Please check all that apply.)

❐ Full-time paid work
❐ Part-time paid work
❐ Homemaker
❐ Unemployed
❐ Receiving Disability
❐ Retired
❐ Other: please specify: ____

What is your marital status?

❐ Single
❐ Married
❐ Widowed
❐ Separated/Divorced
❐ Have a current partner

What is the highest level of schooling that you completed?

❐ Less than 12 Years
❐ High School Graduate or GED
❐ Technical or Trade School
❐ Some College
❐ College Graduate
❐ Graduate School

Do you have a computer at home with daily access to the internet?

❐ No ❐ Yes

Do you have a smart phone or tablet that connects to the Internet, such as an iPhone, Blackberry, or Droid?

❐ No ❐ Yes

Thank you!

Study ID:
Group ID:
Interviewer:
Date:
Time:

Patient & Caregiver Interview Guide Phase I HEART Study

- **Study Overview**
- **Consenting/Questions/Parking & Giftcards**
- **Overview of Session (length, audiotape, confidentiality)**
- **Complete Brief Clinical Survey (diagnosis and treatment information)**

Thank you for being here today to share your experiences as we develop survivorship programs for families at the end of treatment. In this project, we are focused on understanding how we can help caregivers, the family members and friends who provide support to loved ones with head and neck cancer. While we know families face many challenges after finishing treatment, in this project we are specifically focused on nutrition and how we can provide support to caregivers around nutrition in the month after completing treatment.

Begin Recording

1. Let's start by talking about your experiences as you finished your treatment for head and neck cancer. [Refer to clinical survey questions]. You completed all of your treatment in [insert timing] and had [insert treatment types].
    - Patient:
        - How were you feeling at the end of treatment?
        - What were the main physical and emotional challenges you were experiencing at this time?
    - Caregiver:
        - And how were you doing at this time? Please describe any worries or concerns you had at the end of treatment in your role as a caregiver.

2. Let's now discuss nutrition at the end of treatment.
    - Patient & Caregiver:
        - Please describe any nutritional challenges you had at the end of treatment. Please describe your ability to eat and any worries or concerns you had about nutrition at this time.
        - What were your expectations for your/his-her nutritional abilities at this time and in the future?
    - Caregiver:
        - As a caregiver, what, if any, specific concerns did you have about nutrition and about your role as a caregiver to support nutritional recovery?
        - And what were the main things you did to try to support his/her nutritional wellness?

3. Now let's talk about your interactions with your doctors and other health care providers at the end of treatment about nutrition.

Study ID:
Group ID:
Interviewer:
Date:
Time:

- Patient & Caregiver:
    - Did you talk to your providers about nutrition? If yes, when, with which providers and what did you discuss?
    - Did you receive any written materials, referrals or other resources about nutrition? Please describe.
- Caregiver:
    - What about your needs as a caregiver? Can you describe any discussions you had with the healthcare team about questions or concerns you had as a caregiver so you could support his/her nutritional recovery?
    - Please describe how your needs as a caregiver were or were not addressed in the area of nutrition.

4. OK this has been very helpful. Thinking back, what would have been helpful to you at the end of your treatment to help improve your nutritional recovery (patient)/your ability to help with his/her nutritional recovery (caregiver)?
    - Probe about information needs, communication, resources, care coordination, conflicting information, family support, symptom management as raised.

Stop Transcription (note time)

5. Now let's switch gears and I'd like to tell you about a program we are developing to support head and neck cancer patients and their caregivers at the end of treatment. At the end of treatment, our patients and their caregivers will meet with a nurse for a follow-up visit. We will ask them to use a tablet computer to answer questions about their nutritional wellness, symptoms, worries and concerns and about how we can best help them to prepare for their recovery. This information will be used to create a personalized care plan that the nurse will go over with the patients and caregivers. [Show HEART Tool demo screens and care plan example].
    - What are you initial thoughts?
    - What do you like or dislike about the HEART tool (the look and feel of the tablet)?
    - Do you have any specific comments about our plans?
        - Probe regarding comfort using Ipad, important questions to ask at this time, caregiver-specific questions, preferred timing for visit.

6. The final piece of the HEART program is to send caregivers home with support in the form of mobile messages and tips that you can access on your phone or computer. This would prompt patients to report their symptoms and caregivers to report how they are doing a few times a week and then based on their needs, they would be sent messages and tips. [Show sample mobile messages and screens and discuss reactions and suggestions].

Thank you so much for taking the time to meet with us today and help us plan this program. Do you have any final thoughts about our plans to develop this program?
[Complete 2nd page of survey with ratings].
