# Supplementary material for: Development of a Mobile App to Support Head and Neck Cancer Caregiving: Mixed Methods Study
Source: JMIR Cancer. 2025 Jun 10;11:e66471. doi: 10.2196/66471 (PMC12172808; doi:10.2196/66471)
Supplement: Checklist 1 [file cancer-v11-e66471-s002.docx]

| **Guideline** | **Section: page** |
| --- | --- |
| Describe the justification for using a mixed methods approach to the research question | Methods: p 5 |
| Describe the design in terms of the purpose, priority and sequence of methods | Methods: p 5 |
| Describe each method in terms of sampling, data collection and analysis | Data collection: p 6-7 dietitians and p 7-8 dyads  Data analysis: p 8-9 |
| Describe where integration has occurred, how it has occurred and who has participated in it | Methods: p 8-9 |
| Describe any limitation of one method associated with the present of the other method | Strengths and limitations: p 18 |
| Describe any insights gained from mixing or integrating methods | Data Synthesis: App Development p 18  Strengths and Limitations: p 18 |

*[47] O'Cathain A, Murphy E, Nicholl J. The quality of mixed methods studies in health services research. J Health Serv Res Policy. 2008;13(2):92-98.*
